# Supplementary material for: Positive Associations between Body Mass Index and Hematological Parameters, Including RBCs, WBCs, and Platelet Counts, in Korean Children and Adolescents
Source: Children (Basel). 2022 Jan 14;9(1):109. doi: 10.3390/children9010109 (PMC8774222; doi:10.3390/children9010109)
Supplement: Supplementary file 1 [file children-09-00109-s001.zip › children-1478035-supplementary.pdf]

Supplementary Files

Supplementary Table S1. Clinical characteristics of the study participants by weight and sex (n = 7997)

	All participants				Boys			Girls			Statistical differences between boys and girls		
	NW (n = 6421)	OW (n = 782)	OB (n = 794)	P	NW (n = 3350)	OW (n = 443)	OB (n = 466)	NW (n = 3071)	OW (n = 339)	OB (n = 328)	P_a	P_b	P_c
Boys	3350	443	466	< 0.001									
Age (y)	14.33 ± 2.51	14.24 ± 2.51	14.78 ± 2.54	< 0.001	14.35 ± 2.51	14.08 ± 2.49	14.56 ± 2.55	14.31 ± 2.51	14.44 ± 2.52	15.09 ± 2.50	0.498	0.042	0.004
Height SDS	0.17 ± 1.03	0.47 ± 1.04	0.53 ± 1.11	< 0.001	0.18 ± 1.04	0.55 ± 0.98	0.62 ± 1.07	0.16 ± 1.02	0.37 ± 1.11	0.40 ± 1.17	0.431	0.015	0.007
Weight SDS	–0.31 ± 0.89	1.29 ± 0.49	2.20 ± 0.74	< 0.001	–0.33 ± 0.90	1.31 ± 0.48	2.23 ± 0.74	–0.28 ± 0.87	1.26 ± 0.49	2.16 ± 0.73	0.043	0.461	0.160
BMI SDS (kg/m²)	–0.59 ± 0.88	0.86 ± 0.45	1.64 ± 0.75	< 0.001	–0.65 ± 0.88	0.87 ± 0.38	1.62 ± 0.65	–0.52 ± 0.86	0.85 ± 0.53	1.68 ± 0.88	< 0.001	0.962	0.278
WC SDS	–0.49 ± 0.85	1.32 ± 0.18	2.40 ± 0.67	< 0.001	–0.53 ± 0.87	1.32 ± 0.18	2.41 ± 0.65	–0.45 ± 0.83	1.32 ± 0.18	2.39 ± 0.68	< 0.001	< 0.001	0.673
SBP (mmHg)	105.65 ± 9.77	109.94 ± 10.28	113.68 ± 10.95	< 0.001	107.53 ± 10.09	112.69 ± 10.29	116.15 ± 10.73	103.60 ± 8.96	106.35 ± 9.10	110.18 ± 10.29	< 0.001	0.128	< 0.001
DBP (mmHg)	65.59 ± 8.91	66.88 ± 8.88	69.07 ± 9.19	< 0.001	65.82 ± 9.48	67.29 ± 9.75	69.45 ± 9.58	65.33 ± 8.25	66.34 ± 7.58	68.53 ± 8.59	0.028	0.527	0.157
WBC (×10³/mm³)	6.16 ± 1.49	6.65 ± 1.58	7.16 ± 1.65	< 0.001	6.13 ± 1.45	6.68 ± 1.53	7.13 ± 1.60	6.18 ± 1.53	6.61 ± 1.64	7.22 ± 1.71	0.163	< 0.001	0.432
RBC (×10⁶/mm³)	4.81 ± 0.39	4.89 ± 0.40	4.93 ± 0.41	< 0.001	5.02 ± 0.34	5.10 ± 0.34	5.13 ± 0.33	4.59 ± 0.31	4.62 ± 0.30	4.63 ± 0.31	< 0.001	< 0.001	< 0.001
Hemoglobin (g/dL)	13.97 ± 1.23	14.07 ± 1.22	14.19 ± 1.30	< 0.001	14.60 ± 1.11	14.64 ± 1.15	14.79 ± 1.20	13.28 ± 0.96	13.33 ± 0.85	13.34 ± 0.92	< 0.001	< 0.001	< 0.001
Hematocrit (%)	41.86 ± 3.40	42.28 ± 3.42	42.65 ± 3.53	< 0.001	43.43 ± 3.30	43.73 ± 3.38	44.18 ± 3.34	40.15 ± 2.60	40.38 ± 2.42	40.49 ± 2.50	< 0.001	0.431	< 0.001
Platelet (×10³/mm³)	282.96 ± 58.58	296.81 ± 59.92	309.03 ± 63.53	< 0.001	278.59 ± 58.48	295.33 ± 61.62	302.91 ± 61.66	287.73 ± 58.32	298.74 ± 57.65	317.73 ± 65.21	< 0.001	0.133	0.001
Glucose (mg/dL)	90.01 ± 7.39	91.83 ± 11.05	92.45 ± 12.16	< 0.001	90.69 ± 6.89	92.89 ± 13.37	92.53 ± 6.71	89.26 ± 7.84	90.44 ± 6.69	92.34 ± 17.16	< 0.001	0.001	0.851
T-C (mg/dL)	158.25 ± 26.27	163.00 ± 28.46	169.35 ± 29.50	< 0.001	153.81 ± 25.90	161.35 ± 29.78	168.12 ± 30.10	163.10 ± 25.81	165.17 ± 26.52	171.09 ± 28.57	< 0.001	0.059	0.163
HDL-C (mg/dL)	52.16 ± 9.94	47.68 ± 8.63	44.99 ± 8.37	< 0.001	51.13 ± 9.93	46.10 ± 8.33	44.27 ± 7.98	53.27 ± 9.82	49.75 ± 8.59	46.01 ± 8.80	< 0.001	< 0.001	0.004
TG (mg/dL)	79.29 ± 41.79	99.34 ± 54.29	112.75 ± 58.76	< 0.001	76.49 ± 42.07	103.29 ± 58.81	110.92 ± 58.64	82.33 ± 41.26	94.17 ± 47.33	115.34 ± 58.91	< 0.001	0.017	0.297
LDL-C (mg/dL)	90.24 ± 22.42	95.46 ± 25.07	101.81 ± 25.63	< 0.001	87.38 ± 22.08	94.59 ± 26.15	101.67 ± 26.28	93.36 ± 22.38	96.58 ± 23.57	102.02 ± 24.72	< 0.001	0.264	0.849
Alcohol use	1564 (24.36%)	188 (24.04%)	237 (29.85%)	0.003	888 (26.51%)	110 (24.83%)	142 (30.47%)	676 (22.01%)	78 (23.01%)	95 (28.96%)	< 0.001	0.613	0.705
Smoker	718 (11.18%)	88 (11.25%)	106 (13.35%)	0.191	524 (15.64%)	61 (13.77%)	77 (16.52%)	194 (6.32%)	27 (7.96%)	29 (8.84%)	< 0.001	0.015	0.002
Household income ≤ 1st quartile	687 (10.70%)	82 (10.49%)	95 (11.96%)	0.531	362 (10.81%)	39 (8.80%)	50 (10.73%)	325 (10.58%)	43 (12.68%)	45 (13.72%)	0.804	0.102	0.243
Rural residence	985 (15.34%)	113 (14.45%)	125 (15.74%)	0.757	518 (15.46%)	60 (13.54%)	69 (14.81%)	467 (15.21%)	53 (15.63%)	56 (17.07%)	0.803	0.471	0.445
Physical activity	2368 (36.88%)	279 (35.68%)	316 (39.80%)	0.194	1351 (40.33%)	171 (38.60%)	183 (39.27%)	1017 (33.12%)	108 (31.86%)	133 (40.55%)	< 0.001	0.061	0.773
Hypertension	1 (0.02%)	1 (0.13%)	1 (0.13%)	0.124	1 (0.03%)	1 (0.23%)	0 (0%)	0 (0%)	0 (0%)	1 (0.3%)	> 0.999	> 0.999	0.860
T2DM	0 (0%)	0 (0%)	0 (0%)	> 0.999	0 (0%)	0 (0%)	0 (0%)	0 (0%)	0 (0%)	0 (0%)	> 0.999	> 0.999	> 0.999
Dyslipidemia	0 (0%)	0 (0%)	0 (0%)	> 0.999	0 (0%)	0 (0%)	0 (0%)	0 (0%)	0 (0%)	0 (0%)	> 0.999	> 0.999	> 0.999

SDS, standard deviation score; BMI, body mass index; WC, waist circumference; SBP, systolic blood pressure; DBP, diastolic blood pressure; WBC, white blood cell; RBC, red blood cell; T-C, total cholesterol; HDL-C, high-density lipoprotein cholesterol; TG, triglycerides; LDL-C, low-density lipoprotein cholesterol; T2DM, type 2 diabetes mellitus; $P_{a;}$ statistical difference between normal weight boys and girls, $P_{b;}$ statistical difference between overweight boys and girls, $P_{c:}$ statistical difference between obese boys and girls
